# Structural analysis of VirD4 a type IV ATPase encoded by transmissible plasmids of *Salmonella enterica* isolated from poultry products

**DOI:** 10.3389/frai.2022.952997

**Published:** 2022-09-13

**Authors:** Kuppan Gokulan, Sangeeta Khare, Steven L. Foley

**Affiliations:** Division of Microbiology, National Center for Toxicological Research, U.S. Food and Drug Administration, Jefferson, AR, United States

**Keywords:** type IV secretion system, ATPases, *S. enterica*, transmissible plasmids, ligand docking, homology modeling, secretary mechanism

## Abstract

Bacterial species have evolved with a wide variety of cellular devices, and they employ these devices for communication and transfer of genetic materials and toxins. They are classified into secretory system types I to VI based on their structure, composition, and functional activity. Specifically, the bacterial type IV secretory system (T4SS) is a more versatile system than the other secretory systems because it is involved in the transfer of genetic materials, proteins, and toxins to the host cells or other bacterial species. The T4SS machinery is made up of several proteins with distinct functions and forms a complex which spans the inner and outer membranes. This secretory machinery contains three ATPases that are the driving force for the functionality of this apparatus. At the initial stage of the secretion process, the selection of substrate molecules and processing occurs at the cytoplasmic region (also known as relaxosome), and then transfer mechanisms occur through the secretion complex. In this process, the VirD4 ATPase is the first molecule that initiates substrate selection, which is subsequently delivered to the secretory machinery. In the protein data bank (PDB), no structural information is available for the VirD4 ATPase to understand the functional property. In this manuscript, we have modeled VirD4 structure in the Gram-negative bacterium *Salmonella enterica* and described the predicted functional importance. The sequence alignment shows that VirD4 of *S. enterica* contains several insertion regions as compared with the template structure (pdb:1E9R) used for homology modeling. In this study, we hypothesized that the insertion regions could play a role in the flexible movement of the hexameric unit during the relaxosome processing or transfer of the substrate.

## Introduction

Secretion is a central biological process in living organisms, which facilitates the transfer of chemicals, molecules, and toxins across the cell membrane. Bacterial species use multiple secretory apparatuses to facilitate the translocation of several molecules into the host cells, which helps bacterial survival and communication with other organisms in the surrounding environments (Schroder and Lanka, 2005; Fronzes et al., 2009). To date, six types of secretory systems (types I, II, III, IV, V, and VI) have been identified and characterized in the microbial world. Among these systems, the type IV secretion system (T4SS) is the most versatile, which facilitates various functions and has been observed in both Gram-positive and Gram-negative bacteria. The T4SS shares several structural and functional features with bacterial conjugation systems. The functions of T4SS in bacterial species include (1) translocation of proteins or toxins to the host cell, (2) horizontal transfer of plasmid DNA between bacteria during conjugation, and (3) uptake and release of DNA molecules that allow the exchange of DNA with the extracellular environment or host cells (Grohmann et al., 2003; Wallden et al., 2010).

The presence of T4SS machineries has been documented in several pathogenic bacteria that include *Helicobacter pylori, Streptococcus suis, Bordetella pertussis, Brucella* spp., and *Legionella pneumophila* (Kwok et al., 2007; Zhao et al., 2011). These bacterial species employ T4SS to inject virulence factors into host cells (Corbel, 1997; Ninio and Roy, 2007). Most of the studies elucidating *Salmonella* pathogenicity have been focused on serovar Typhimurium; however, there is a knowledge gap in understanding how different serovars lead to infection and whether putative virulence factors located on plasmids impact the ability of *Salmonella* to infect different hosts; for example, how *Salmonella enterica* isolates containing T4SS differ from those that lack T4SS. Recent CDC data show that non-typhoidal *Salmonella* is the leading cause of diarrhea globally, which accounts for roughly 153 million gastroenteritis cases and 57,000 deaths annually (Healy, 2020). The mode of transmission occurs *via* the consumption of contaminated food products including animal-derived products, seafood, fresh produce, and fruits (Mellou et al., 2021). *S. enterica* serovar Heidelberg is another leading serovar that mostly infects poultry (turkey and chicken) and is a major cause of severe illness in humans through the consumption of contaminated poultry products. *S*. Heidelberg strains are often resistant to several antimicrobial agents, and surveillance data show that drug-resistant strains are on the rise. National Antimicrobial Resistance Monitoring System (NARMS) data show that the percentage of *S*. Heidelberg isolates from human and poultry that are resistant to cephalosporin has been on the rise (Winokur et al., 2000) and correlates with the spread of AmpC β-lactamase. This β-lactamase is encoded by *bla*_CMY_ genes and is linked with transmissible plasmids. Studies have shown that *S*. Heidelberg harbor plasmids are able to transfer genes and are also responsible for multidrug resistance and virulence.

Studies have shown that certain *S. enterica* strains isolated from food-animal sources harbor transmissible plasmids (Johnson et al., 2010). In addition, multiple isolates have been shown to have transmissible plasmids that harbor T4SS encoding genes in *S. enterica* (Han et al., 2012). Moreover, the importance of T4SS encoding genes in bacterial invasion and virulence of *S. enterica* on macrophage infection was demonstrated by our group (Gokulan et al., 2013). In Gram-negative bacteria, the T4SS core complex is composed of 12 proteins (VirB1 to VirB11 and VirD4) that span across both inner and outer transmembrane domains to facilitate secretion. The T4SS complex is further divided into three groups: (i) scaffold with translocation channel; (ii) ATPases, and (iii) pilus. The plasmid sequence analysis of *S. enterica* revealed that the presence of the VirB/D4 T4SS core complex is similar to the *Agrobacterium tumefaciens* VirB/D T4SS. The sequence analysis also revealed the absence of the VirB7 sequence in the core complex in *S. enterica*. This finding was consistent with whole-genome sequence results of 44 outbreak strains of *S*. Heidelberg isolates (animal, retail meats, and human clinical isolates) that revealed the presence of transmissible plasmids that encode T4SS in 21 isolates (Hoffmann et al., 2013).

The T4SS inner membrane complex (ATPases system) contains three ATPase proteins (i.e., VirD4, VirB4, and VirB11), which are the driving force for the assembly of T4SS, substrate transfer that can include virulence factors. The VirB4 crystal structure bound with ADP has been reported elsewhere (Wallden et al., 2012). VirB4 is a highly conserved protein in the T4SS machinery and is composed of N-terminal and C-terminal domains. There is no structural information available for VirD4 of *S. enterica*; therefore, this study was undertaken to understand the structural and functional details of VirD4. In this study, we employed bioinformatics tools for the homology modeling of the T4SS machinery of *S. enterica* to understand the functional aspects of the VirD4 ATPases.

## Methods

### *Salmonella* strain and sequence selection for homology modeling

The VirD4 sequence used for homology modeling was derived from *S. enterica* strain 163 (which was isolated from an infected turkey) (pSH163_34, GenBank accession No. JX258656). Protein sequences were determined with the RAST annotation pipeline (Argonne National Laboratory, Chicago, IL, USA), and the protein identities were determined by BLAST comparisons to GenBank.

### Secondary structure and protein structural fold prediction

VirD4 protein sequences were submitted to the protein fold reorganization server to predict structural folding based on the sequence and similarity in protein folding (www.sbg.bio.ic.ac.uk/phyre2) and the Swiss model (swissmodel.expasy.org) (Soding, 2005; Waterhouse et al., 2018). We compared the predicted structures from these programs and selected the best fit secondary structure and protein fold conserved template for homology model building. To analyze the confidence of the predicted model, we used sequence identity, similarity, secondary structure prediction, and structural superposition for conservation of protein fold and obtained root mean square deviation (RMSD) value during structural alignment and *Z*-score value (DALI search) (Holm and Rosenstrom, 2010). The final model was energy minimized using SYBYL (www.tripos.com), which was further inspected using WINCOOT (www.ysbl.york.ac.uk) to see if any clashes occur between side chain residues by comparing with the template model. The stereochemistry of the predicted structure was assessed with the program PROCHECK (www.ebi.ac.uk).

### Structural cavity analysis by CASTp

In the PDB, no structural information is available for the VirD4 protein of T4SS machinery; therefore, there is a lack of information about the substrate binding region. In addition, the VirD4 sequence had several insertion regions compared with the template structure (bacterial conjugate coupling protein pdb 1E9R). To predict the nucleotide-binding region and exclude it from the shallow depression, cavity prediction was performed for modeled VirD4 structure. Cavities exhibit an entrance that connects the interior of protein with the outside solution or small molecules. Before submission to cavity prediction, we removed residues 1-106 from the model, which is predicted to be in the transmembrane region. To predict a probable substrate binding site, we initially employed the CASTp program that analyzes the topology of the structure and predicts the concave cavities, surface area, and location (Binkowski et al., 2003). In addition, we also used the protein structural fold search engine to identify the functional site in the homology model. The CASTp predicted concave cavity location, and the functional site predicted by the protein structural fold search engine was further validated by docking the nucleotide at the active site.

### Ligand docking by CB-DOCK and 3D-ligand docking method

Based on the cavity prediction and functional site prediction, ligand docking was performed by CB-DOCK and 3D-ligand docking for validation (Liu et al., 2020). The VirD4 homology model was converted into pdbqt format for docking purposes. VirD4 ATPase initiates protein assembly and facilitates the secretion of toxins into the host cells in association with partner proteins. Therefore, an ADP ligand was generated and converted into an SDF file format for docking.

### Hexameric structure

The fundamental functions of the VirD4 protein are to recruit the substrates and then deliver them to the secretion channel. It is also known as coupling protein that contains Walker A and B sequence motifs. These motifs play a major role in nucleotide binding and hydrolysis. The crystal structure of the cytoplasmic region of the P-loop containing nucleoside triphosphate hydrolases (1E9R) assembles to form a hexameric structure. The VirD4 protein displayed 70 to 80% conservation of secondary structure with 1E9R structure for 395 residues. The conservation of secondary structure and structural fold implicates the functional similarity between them. To construct *S. enterica* VirD4 hexameric form, we employed 1E9R hexameric structure as a template and translated modeled VirB4 into each monomer. The hexameric structure was globally energy-minimized using SYBYL. Figures were generated using the program Pymol (www.pymol.org).

## Results

**Figure 1:**
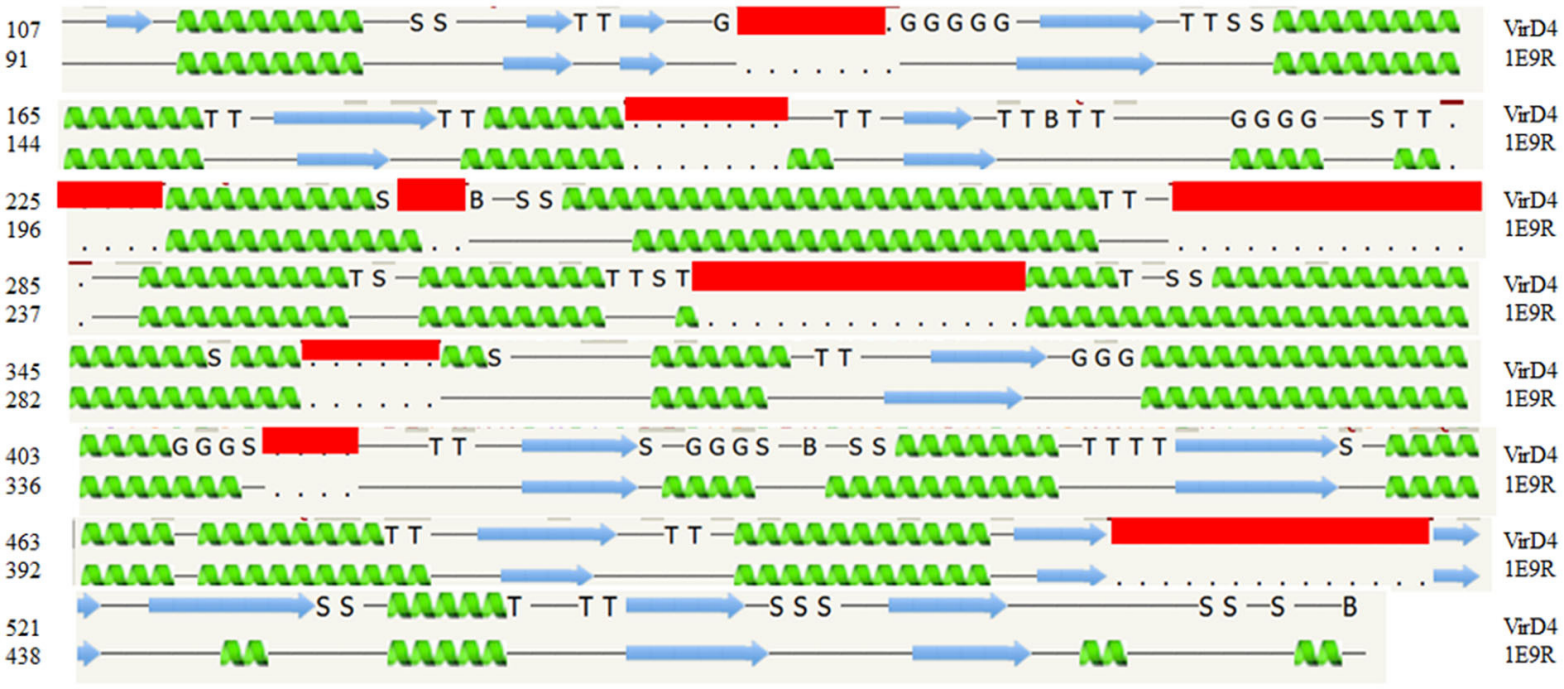
The secondary structure alignment between the template structure (1E9R) and *Salmonella enterica* VirD4 sequence. The secondary structure prediction shows that approximately 400 amino acids are aligned very well between them. The *S. enterica* VirD4 protein sequence has several insertion regions, which are shown in the red box. The α-helix prediction is shown in green, and the β-strand is shown by the blue arrow. In this figure, G-indicates the 3-turn helix, T-indicates the hydrogen-bonded turn, and S indicates the bend. The top row is VirD4 starts with residue 107, and the bottom row is template starts with residue 91(1E9R).

**Figure 2:**
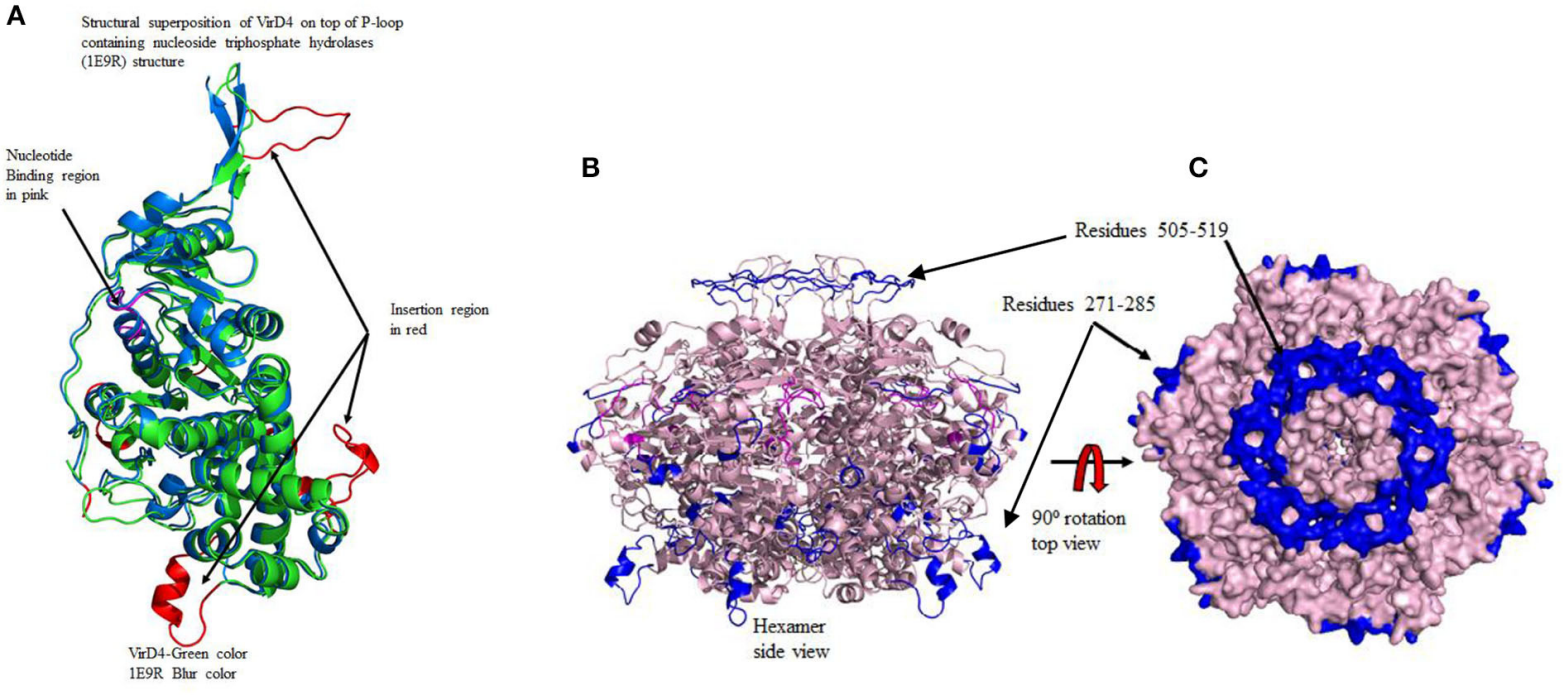
**(A)** The structural superposition of modeled VirD4 structure on top of 1E9R template structure. This diagram also shows the insertion regions and nucleotide binding regions in different colors. Color code: green VirD4 and cyan 1E9R template structure. The two long insertion regions are labeled as Regions I (residues 271-295) and II (residues 505-519). **(B)** The left side of the diagram shows the formation of the hexamer. The nucleotide-binding regions are located at the dimer interface shown in pink. Two long insertion regions are shown in blue, one occupies the top of the hexamer and the other is at the bottom. The transmembrane binding region forms a circular disc to insert into the membrane. **(C)** The right surface diagram shows the top view. In the hexameric form, the opening of the tunnel is much wider near the transmembrane region, whereas, in the cytoplasmic region, the opening is very narrow. The two insertion regions are also shown in blue. At the transmembrane insertion region, all six monomers connected to each other forming a ring-like disc.

The protein structural fold search engine (Phyre2) predicted a few structural coordinates from the PDB based on protein sequence identity and similarity that aided as a template for homology modeling for *S. enterica* VirD4 protein, and specifically, these include P-loop containing nucleoside triphosphate hydrolases (1E9R), Type IV Coupling Complex (T4CC) from *L. pneumophila* (6SZ9), and structure of VirB4 of *Thermoanaerobacter pseudethanolicus* (4AG5) (Pena et al., 2012; Wallden et al., 2012; Meir et al., 2020). The predicted secondary structure of VirD4 protein displayed a high percentage of structure conservation with P-loop containing nucleoside triphosphate hydrolase structure (Pena et al., 2012). The predicted secondary structure of VirD4 protein was found to be around a 70 to 80% match with the template secondary structure (1E9R) for 395 residues. The sequence alignment analysis reveals that the VirD4 sequence of *S. enterica* showed 21% sequence identity and 56% sequence homology with P-loop containing nucleoside triphosphate hydrolase sequence (1E9R). The sequence alignment displayed that the VirD4 protein sequence had several insertion sequences in comparison with template structure (pdb#1E9R) (Figure 1). The protein structural fold search engine also predicted VirB4 of *T. pseudethanolicus* as a template model, which belongs to T4SS (Wallden et al., 2012); however, the secondary structure prediction and structural alignment displayed less conservation in comparison with 1E9R coordinates. Therefore, the 1E9R coordinate was used for homology modeling of the VirD4 structure based on the secondary structure conservation and similarity in the structural fold. Although VirD4 had only 21% sequence identity with 1E9R coordinates, the structural fold was highly similar (Figure 2A). Approximately 70% of the predicted model was built with more than 90% confidence.

For the structural analysis, we deleted the N-terminal transmembrane region of VirD4 protein residues 1-116 and the last 40 amino acids (580-620) from the C-terminal region due to low confidence in the homology model building. Then, we superimposed C-α carbon atoms of the VirD4 homology model on top of 1E9R coordinates for structural analyses. The VirD4 C-α carbon atoms of residues from 107 to 474 were superimposed on top of the template structure (1E9R) and C-α carbon atom residues from 91 to 491 with RMSD 0.6 Å (Figure 2A). The structural alignment revealed that VirD4 structure insertion regions occupy the connecting loop and are located away from the core structure (shown in red color in Figure 2A). Most of the insertions are between 4 and 6 residues except two regions (Table 1). Two insertion regions were around 10 to 15 residues long, and one insertion was positioned in the connecting loop at the bottom of the hexamer (Figure 2A shown in red color). The second region occupies the top of the core structure (left side cartoon diagram shown in blue in Figure 2B) that forms a donut-like structure highlighted in blue (right side surface diagram shown in Figure 2C). The homology model was minimized, and the quality of the structure was inspected for clashes, rotamers, and amino acid geometry (Ramachandran plot), and all were in acceptable ranges. We also generated a hexameric form of the VirD4 model, which forms a ring-like structure (Figure 2B). The final VirD4 homology model was submitted to DALI search for structural alignment prediction from the PDB. The DALI search predicted several structures from the PDB; however, the 1E9R structure was the top-most structure with a *Z*-score of 37.4%, with a low RMSD, and a clear separation from the remaining predicted structures which all had very low Z-scores with higher RMSD.

**Table 1:** The position of the insertion region and the number of residues in each region.

**VirD4 Insertion region**	**Residues**
136–142	KDKKIIR
189–195	SLIRKVI
224–228	SEGFN
271–285	NDKAGLKTLDIEPV
312–325	SELRGKTLADI
355–360	ANPNVA
411–415	MPTD
505–519	TIGSKSKSRSRGGTS

**Figure 3:**
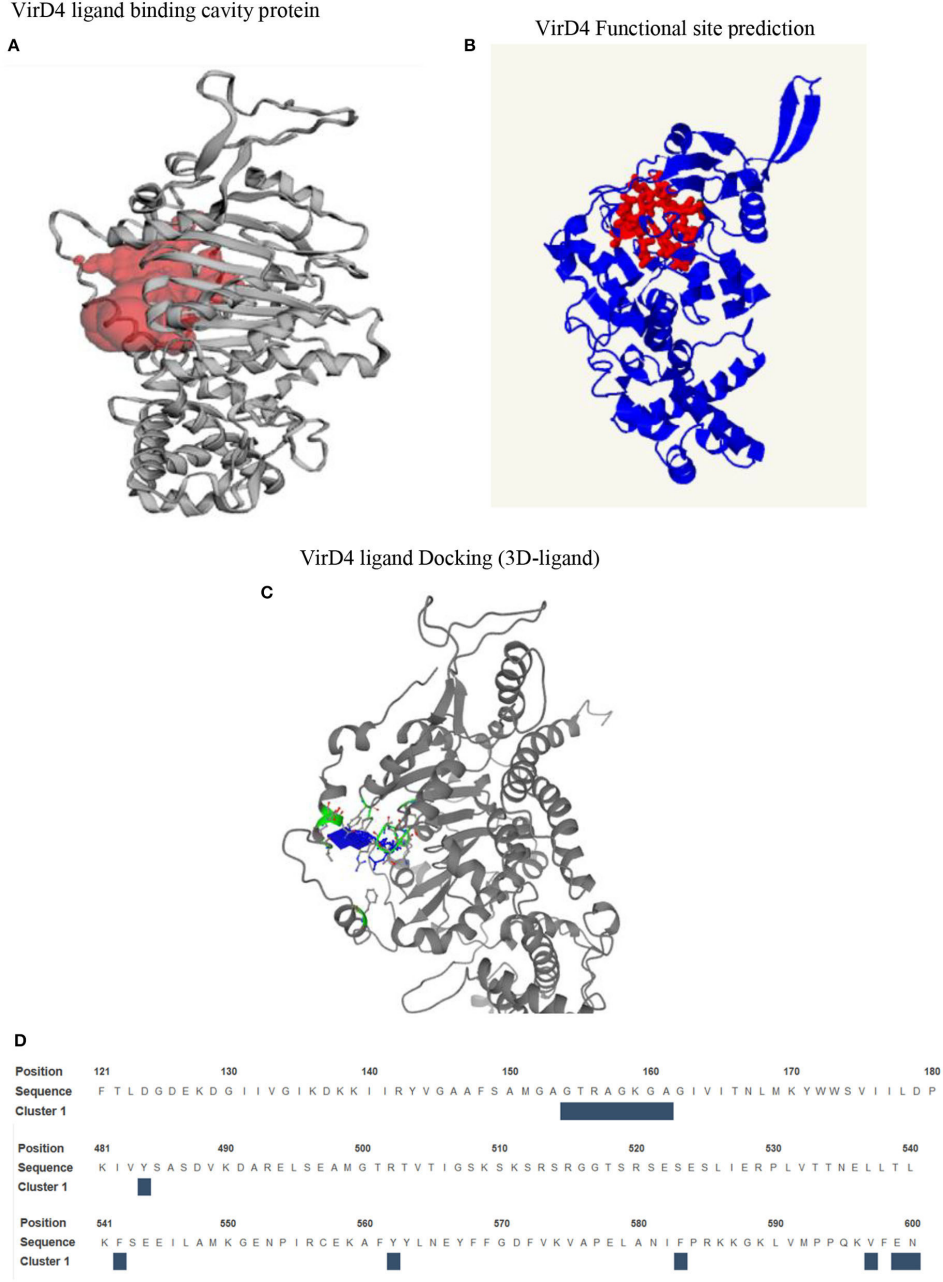
**(A)** Surface topology predicts the cavity on VirD4 structure. In this figure, we have shown the well-defined cavity that occupies the N-terminal region between the β-strands and is surrounded by α-helices. **(B)** The prediction of functional residues that are likely involved in substrate binding. **(C)** The nucleotide-binding region is shown in blue, which is surrounded by Walker A and B motifs, shown in green with stick representation. **(D)** This sequence shows the location of Walker A and B motifs. The interacting residues are shown above the blue box.

The homology structure of VirD4 contains two domains that include an α-helical domain and β-strands surrounded by α-helices or a nucleotide binding region, which is very similar to the 1E9R structure (Figure 2A). The sequence alignment shows that the C-terminal region contains highly conserved amino acids as compared with the N-terminal region. Earlier it was shown that VirD4 is essential machinery in first recruiting the substrate and subsequently transferring the substrate to VirB11; therefore, VirD4 is known as a coupling protein. Due to its role in secretory pathways, we analyzed the functional property of VirD4 protein structure by analyzing surface topology to predict a cavity, which could be a probable nucleotide-binding pocket (CASTp server). The surface topology characterization predicted two pockets in the VirD4 structure with a surface area of 1,395 Å (Pocket-1) and 2,539 Å (Pocket-2) (Figure 3A). The boundary of Pocket-1 is well-defined with a concave surface located in the region of β-strands surrounded by α-helices, whereas the predicted Pocket-2 boundary is scattered and occupies a larger surface area. We also analyzed the functional sites of the VirD4 structure of *S. enterica* using a 3D ligand docking server and predicted the probable nucleotide-binding region (Figure 3B, which is shown in red color). 3D-ligand docking server predicted 5 binding clusters, and among them, clusters 3 and 5 predicted the Walker A and B motifs; however, predicted cluster 3 was found to be a more accurate and superimposed well with the earlier solved crystal structure. The 3D-ligand predicted cluster and CB-DOCK predicted nucleotide binding site matched well with Pocket-1 predicted by CASTp. The ligand binding cavity is surrounded by several charged residues with interacting distances, and these residues overlap with functional site residues identified by CASTp, CB-DOCK, and 3D-ligand dock predicated region (Figure 3C). The 3D-ligand docking predicts the probability of active site residues (probability score 0.33 or above of a residue is considered involving in binding) that are involved in ligand binding. Table 1 shows the list of residues that can participate in nucleotide binding and shows the solvent accessibility of these residues. In addition, the table provides the conservation information of the binding residues. The 3D-ligand cluster prediction was based on the binding of 15 ligands (ADP: 5, SO4: 7, and GNP: 3) (Figure 3D).

**Figure 4:**
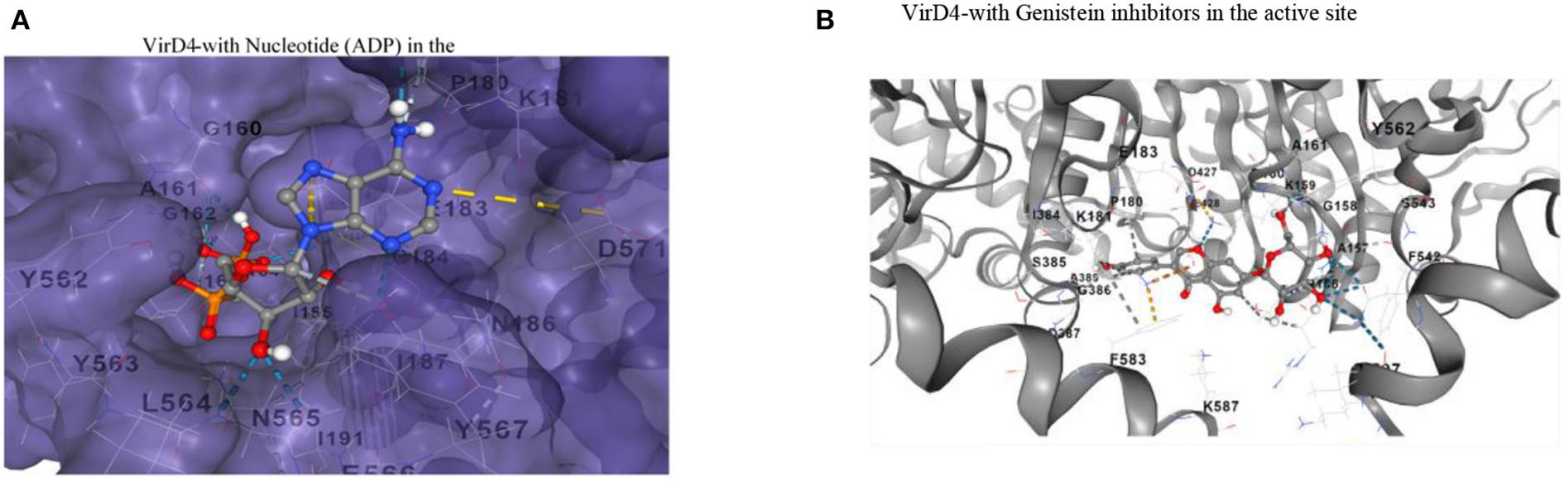
**(A)** The substrate binding pocket in which the ADP molecule is docked in the active site and represented in the stick model. The interacting residues are shown and labeled in the active site cavity. The α-and β-phosphate groups of ADP are surrounded by P-loop binding residues. The adenine and ribose molecules are interacting with several charged residues. **(B)** Binding of genistein in the active site cavity of VirD4 structure on docking. The interacting residues are very similar for both ADP and genistein. Part of the molecule is surrounded by Walker A structure, a very similar phosphate group of ADP. Walker A and B motifs are mostly involved in interacting with genistein.

Then, we analyzed conserved residues of the active site in comparison with other ATPases of bacterial secretory systems. Like other ATPases, VirD4's predicted functional site residues possess conserved nucleotide binding motifs (Walker A and B motifs, Supplementary Table 1). We also analyzed the presence of Walker A and B motifs from other related ATPases as well. The result shows that these motifs are highly conserved and aligned with other bacterial ATPases that include the family of conjugative coupling factor, conjugation transfer system, and TraN, a hallmark protein of the F-type IV secretion system (Supplementary Table 2). The docked ligand is surrounded by Walker A and B motif sequence indicating the functional role as an ATPase (Figure 4A). This docked conformation is positioned to interact with several conserved residues at the active site, and they are comparable with nucleotide-bound VirB4 and other ATPases (Wallden et al., 2012). The phosphate groups of ADP ligand are surrounded by residues of Gly-Thr-Arg-Ala-Gly-Lys-Gly-Ala-Gly-Iso-Val-Iso, Tyr562, and Tyr563 like other ATPases. Specifically, α- and β-phosphates interact with backbone nitrogen amides of the P-loop of the Walker A-motif. Similarly, adenine ring and ribose sugar molecules are surrounded by several charged residues including Lys181, Arg182, Glu183, Asn186, Asn565, and Asp571 positioned within hydrogen bonding distances (Figure 4A). Ligand docking showed several orientations of the ligand with energy minimization scores, and Figure 4A shows the best-fitted ligand with lowest ΔG energy (-8.3 kcal/mol). We also docked genistein (a known RecA inhibitor) at the predicted substrate binding cavity. The docking results show that the 5-hydoxy-3-(4-hydroxyphenyl)-7 group of genistein is surrounded by Walker A motif sequence very similar to phosphate groups of nucleotides (ADP). The hydroxy-phenyl group is similarly occupied to the adenine ring of nucleotide, and interacting residues Pro180, Lys181, and Glu183 are very similar for both nucleotide and genistein (Figure 4B).

## Discussion

The T4SS is large multiprotein machinery spanning the inner and outer membranes of Gram-negative bacteria. This system is more versatile compared with other types of secretion systems and is involved in DNA conjugation between two cells of the same bacterial taxa, injecting proteins or toxins to the host cells or other bacterial species and contributing to the release or uptake of genetic material (Lederberg and Tatum, 1953; Lawley et al., 2003; Backert and Meyer, 2006). The bacterial conjugation system has been linked to multidrug resistance due to horizontal DNA transfer, which poses a threat to human health (Leclercq et al., 1988; Douard et al., 2010). The structural components of the bacterial T4SS are ATPases, channel-forming multi-proteins, and pilus (Alvarez-Martinez and Christie, 2009; Bhatty et al., 2013). Each component arbitrates a specific biological function during the secretion process. T4SS contains three ATPases that include VirB4, VirB11, and VirD4, which are responsible for powering the secretory machinery on binding with nucleotide. The next major structural component is the translocation channel formed by several proteins including VirB3, B6, B7, B8, B9, and B10, which facilitate the translocation of toxins or genetic material to the host cells or other bacteria. The pilus is an extracellular structure located on the outer membrane, and it helps with the adhesion function during interaction with host cells. Characterizing the function of individual plasmid-encoded genes and proteins involved in secretion will provide an improved understanding of the structural basis of antimicrobial resistance and the molecular mechanism of pathogenesis.

In *S. enterica*, VirD4 consists of 640 amino acids, which form N-terminal and C-terminal domains. The amino acid sequences are highly variable at the N-terminal, but C-terminal has more conserved residues like other bacterial VirD4 proteins. The monomeric form retains structural folds very similar to P-loop containing nucleoside triphosphate hydrolase structure (Pena et al., 2012). The only difference is that the VirD4 structure has several insertion regions, and most of them occupy the outer surface of the core structure except amino acids 505-519. An earlier study showed the stoichiometry of VirD4, and it consists of six subunits assembled as a hexamer (Pena et al., 2012). In the generated hexameric form, the insertion region residues 505-519 occupy the top of the core structure and form a donut-like ring structure. In the hexameric form, the insertion region could (a) contribute to attachment with the inner transmembrane, (b) provide more flexibility to interact with a partner protein, and (c) recruit substrates. Likewise, residues 271-285 occupy the outer surface, and the substrate selection is mostly directed by the interaction between the relaxosome and coupling protein. Based on its location of residues 271-285, we proposed that it could contribute to the interaction with relaxosome or VirB11 during the transfer of substrate to the secretary channel. These insertions are absent in P-loop containing nucleoside triphosphate hydrolases. The hexameric structure has a wide opening (20Å) near the transmembrane region, whereas, in the cytoplasmic region, the opening is narrower (14Å). In the hexameric structure, the nucleotide-binding regions are occupied between two monomer interfaces with a wide cavity to enter the nucleotide. This is consistent with earlier reported crystal structures (Wallden et al., 2012). The α- and β-phosphates of ADP are at a favorable distance to interact with backbone amines of the P-loop of the Walker motif, which lines other ATPases. Genistein docking reveals that the 5-hydroxy-3-(4-hydroxyphenyl) group occupies the same location as that of α-and β-phosphates of ADP, and its hydroxyl groups interact with the P-loop of the Walker motif. The genistein binding location and ADP binding location are very similar in the active site cavity. In addition, the interacting residues are also very similar for both ligands. Earlier studies have shown that genistein inhibits RecA ATPase; therefore, we proposed that genistein could probably inhibit VirD4 ATPase as well. However, this hypothesis needs to be experimentally verified.

## Conclusion

**Figure 5:**
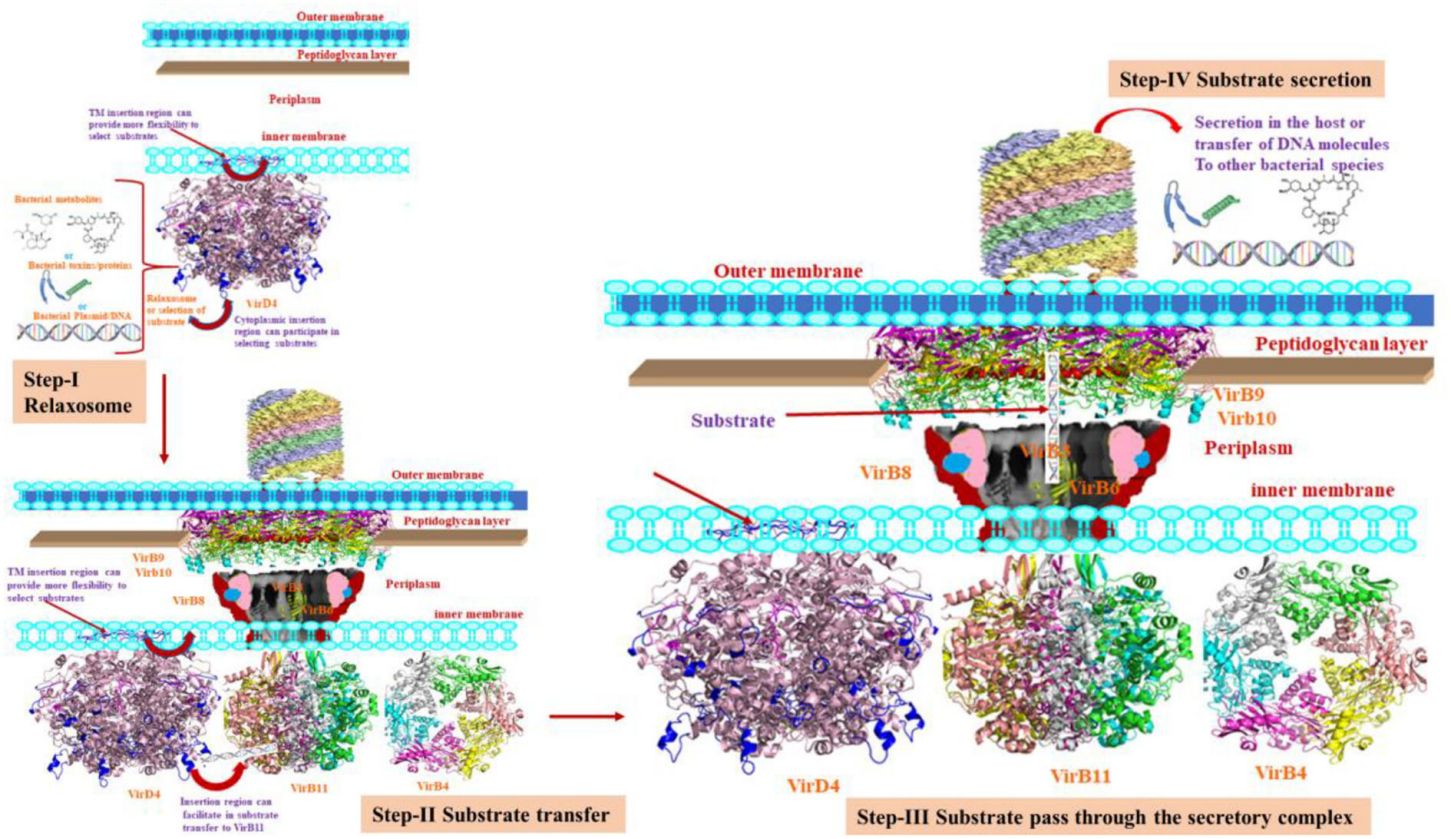
The various steps of the secretion process. Step-I shows the relaxosome where VirD4 recruits the substrate. During this process, the insertion regions may allow flexible movements. Step II shows the transfer of substrate to VirB11. This cartoon also depicts VirD4 possibly interacting with VirB11. Step III shows the translocation of the substrate through the secretory machinery. Step-IV shows the secretory products to the host cells to transfer to bacterial species.

The VirD4 protein had a 21% sequence identity with P-loop containing nucleoside triphosphate hydrolase structure. Although it has several insertion regions, the structural fold is very similar, which indicates the functional and structural conservation between them. Based on the location of two insertion regions, we hypothesized that it could provide more flexibility to interact with partner proteins during substrate transfer to VirB11 (Figure 5). The ligand or inhibitor docking reveals that Walker A and B motifs are involved in ligand binding. The proposed hypothesis needs to be biochemically validated.

## Data availability statement

The original contributions presented in the study are included in the article/Supplementary materials, further inquiries can be directed to the corresponding author.

## Author contributions

Conceptualization: KG and SF. Methodology and analysis: KG and SK. Writing—original draft and writing—review and editing: KG, SK, and SF. All authors contributed to the article and approved the submitted version.

## Conflict of interest

The authors declare that the research was conducted in the absence of any commercial or financial relationships that could be construed as a potential conflict of interest.

## Publisher's note

All claims expressed in this article are solely those of the authors and do not necessarily represent those of their affiliated organizations, or those of the publisher, the editors and the reviewers. Any product that may be evaluated in this article, or claim that may be made by its manufacturer, is not guaranteed or endorsed by the publisher.

## Author disclaimer

This manuscript reflects the views of the authors and does not necessarily reflect those of the U.S. Food and Drug Administration.

## Supplementary material

Supplementary Table 1 Probability scores and conservation values of residues that are involved in ligand binding on docking.

Supplementary Table 2 The Walker A and B motif sequences. The motif search reveals that the Walker A and B motifs are highly conserved in bacterial ATPases that belong to the TRAG family, conjugal transfer proteins, and putative T4SS pathway proteins.
