# Supplementary material for: Structural analysis of VirD4 a type IV ATPase encoded by transmissible plasmids of Salmonella enterica isolated from poultry products
Source: Front Artif Intell. 2022 Sep 13;5:952997. doi: 10.3389/frai.2022.952997 (PMC9513038; doi:10.3389/frai.2022.952997)
Supplement: Supplementary Table 1 — Probability scores and conservation values of residues that are involved in ligand binding on docking. [file Table_1.DOCX]

Supplemental Table I

| Residue | Amino Acid | Conservation (0-1) | Solvent Accessibility | Probability (0-1) |
| --- | --- | --- | --- | --- |
| **154** | **GLY** | **0.59** | **4.27** | **0.97** |
| **155** | **THR** | **0.6** | **81.71** | **1.0** |
| **156** | **ARG** | **0.66** | **107.78** | **1.0** |
| **157** | **ALA** | **0.61** | **0.80** | **1.0** |
| **158** | **GLY** | **0.68** | **16.70** | **1.0** |
| **159** | **LYS** | **0.7** | **51.09** | **1.0** |
| **160** | **GLY** | **0.62** | **28.35** | **1.0** |
| **161** | **ALA** | **0.55** | **26.55** | **0.91** |
| **484** | **TYR** | **0.5** | **0.00** | **0.95** |
| **542** | **PHE** | **0.34** | **32.80** | **0.11** |
| **562** | **TYR** | **0.55** | **155.94** | **0.83** |
| **583** | **PHE** | **0.0** | **87.40** | **0.16** |
| **597** | **VAL** | **0.0** | **97.34** | **0.16** |
| **599** | **GLU** | **0.0** | **56.83** | **0.23** |
| **600** | **ASN** | **0.0** | **60.17** | **0.21** |
| **601** | **GLU** | **0.0** | **92.12** | **0.17** |
