# Supplementary figures and images for: Structural analysis of VirD4 a type IV ATPase encoded by transmissible plasmids of Salmonella enterica isolated from poultry products
Source: Front Artif Intell. 2022 Sep 13;5:952997. doi: 10.3389/frai.2022.952997 (PMC9513038; doi:10.3389/frai.2022.952997)

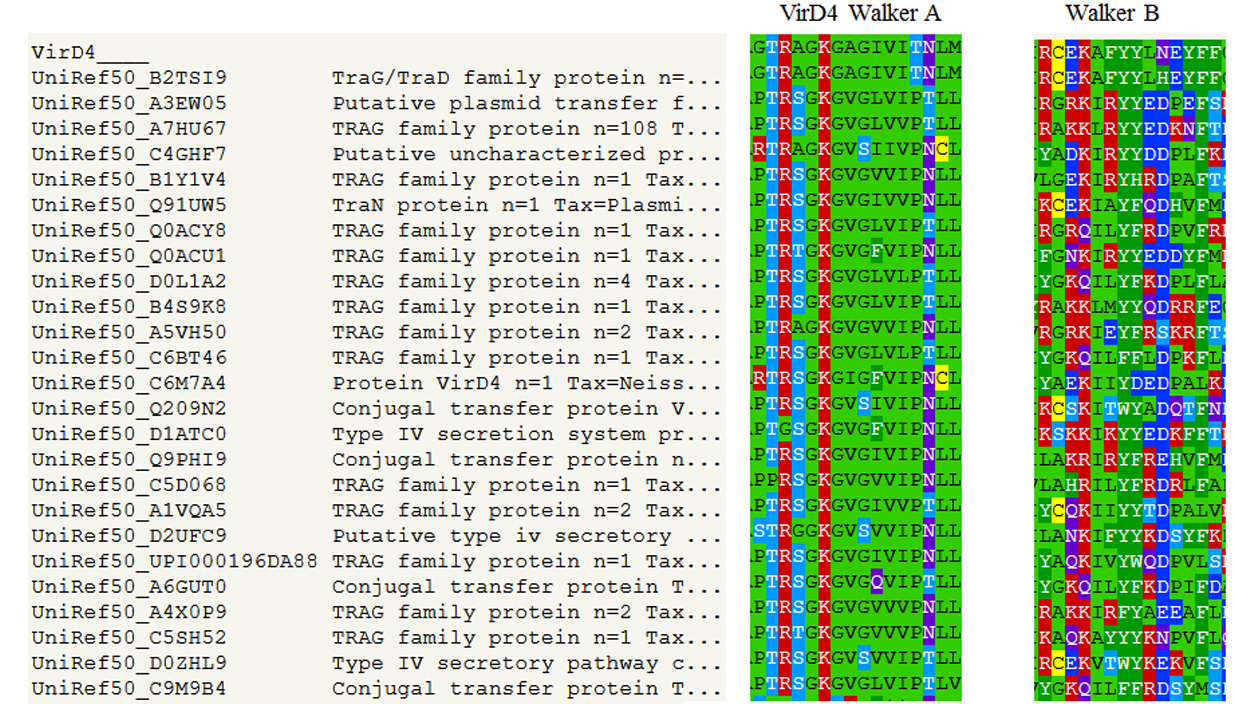

Supplement: Supplementary Table 2 — The Walker A and B motif sequences. The motif search reveals that the Walker A and B motifs are highly conserved in bacterial ATPases that belong to the TRAG family, conjugal transfer proteins, and putative T4SS pathway proteins. [file Image_1.TIF]
